# Establishment and clinical application of a rapid RPA-LFS assay for detecting *Moraxella catarrhalis*

**DOI:** 10.3389/fcimb.2026.1840184

**Published:** 2026-08-11

**Authors:** Jiansheng Lin, Xing Liu, Yinna Wang, Chunyan Lin

**Affiliations:** 1 Microbiology Laboratory, Quanzhou Women’s and Children’s Hospital, Quanzhou, China; 2 Microbiology Laboratory, The Affiliated Women’s and Children’s Hospital of Huaqiao University, Quanzhou, China; 3 Department of Ophthalmology, Quanzhou Women’s and Children’s Hospital, Quanzhou, China; 4 Department of Ophthalmology, The Affiliated Women’s and Children’s Hospital of Huaqiao University, Quanzhou, China; 5 Respiratory Department, Quanzhou Women’s and Children’s Hospital, Quanzhou, China; 6 Respiratory Department, The Affiliated Women’s and Children’s Hospital of Huaqiao University, Quanzhou, China

**Keywords:** clinical application, *copB*, *Moraxella catarrhalis*, rapid, RPA-LFS

## Abstract

**Background:**

*Moraxella catarrhalis* (*M. catarrhalis*) is an important opportunistic pathogen causing respiratory tract infections, and current detection methods are limited by low sensitivity, heavy instrument dependence or complex operation, which cannot meet the needs of rapid clinical and on-site testing.

**Methods:**

The *copB* gene is a species-specific conserved gene of *M. catarrhalis*, which was selected as the target for specific detection. Primers and probe were designed using Primer Premier 5.0 software and online tools. The limit of detection was determined using standard strains, and the clinical applicability of the recombinase polymerase amplification-lateral flow strip assay (RPA-LFS) was evaluated by testing 300 clinical samples. Culture combined with real-time quantitative PCR (qPCR) was applied as the composite reference standard to evaluate clinical diagnostic performance.

**Results:**

The optimized RPA-LFS assay was incubated at 37 °C for 15 minutes, which specifically detected all 20 *M. catarrhalis* strains without cross-reactivity with other pathogens, and its limit of detection reached 13.4 CFU/mL without interference from heterologous bacterial DNA. The clinical sensitivity and specificity of RPA-LFS were 96.26% and 88.08%, respectively.The positive detection rate of the assay in clinical specimens was 42%, significantly higher than that of PCR (15%) and conventional culture (9%).

**Conclusion:**

The established *copB*-based RPA-LFS assay is rapid, simple, instrument-independent, highly sensitive and specific, with excellent clinical detection performance, which can serve as an effective technical tool for the rapid and on-site detection of *M. catarrhalis* and support early clinical diagnosis of its infections.

## Introduction

*Moraxella catarrhalis* (*M. catarrhalis*) is a Gram-negative diplococcus belonging to the genus *Moraxella* (Verghese and Berk, 1991). It is widely colonized in the human upper respiratory tract and was once regarded as a non-pathogenic commensal bacterium. However, it has been increasingly recognized as an important opportunistic pathogen responsible for lower respiratory tract infections and systemic infectious diseases, posing a substantial health threat to children, the elderly, and immunocompromised individuals (Verduin et al., 2002; Blakeway et al., 2017). Historically, *M. catarrhalis* was assigned to the genus *Branhamella* in 1970 and later reclassified as a subgenus of *Moraxella* in 1984, designated *Moraxella* (*Branhamella*) *catarrhalis* (Catlin, 1972; Bovre, 1984). At present, *M. catarrhalis* remains the most widely accepted nomenclature (Verduin et al., 2002).

The colonization rate of *M. catarrhalis* exhibits a significant negative correlation with age. Preschool children carry a high carriage rate of up to 54% and represent the primary high-risk population for pediatric infections (Ejlertsen et al., 1994; Belachew et al., 2023). In contrast, the nasopharyngeal colonization rate in healthy adults is extremely low, ranging from 1% to 3% (DiGiovanni et al., 1987). Nevertheless, colonization rates are markedly elevated in patients with chronic lung diseases, immunodeficiency, and advanced age, rendering these groups important reservoirs for transmission (Verduin et al., 2002). *M. catarrhalis* ranks as the third most common pathogen of respiratory tract infections, preceded only by *Haemophilus influenzae* and *Streptococcus pneumoniae*. Notably, the isolation rate of *M. catarrhalis* is considerably higher in autumn and winter than in spring and summer (Verduin et al., 2002).

Pneumonia caused by *M. catarrhalis* is a common and frequently encountered disease in children, characterized clinically by high fever, dyspnea, tachycardia, tachypnea, and productive cough, often resulting in prolonged hospital stays. Besides pneumonia, *M*. *catarrhalis* is a major pathogen of acute otitis media in children, and also a key trigger for acute exacerbation of chronic obstructive pulmonary disease (COPD) in adults (Perez and Murphy, 2017; Murphy et al., 2019). Conventional culture methods suffer from low sensitivity, long turnaround times (24–48 hours), and susceptibility to antibiotic interference, thus failing to satisfy the demand for rapid and accurate diagnosis. With high sensitivity and high specificity, molecular detection techniques have become the mainstream approaches for *M. catarrhalis* detection, including nucleic acid amplification, and mass spectrometry (Greiner et al., 2003; Schaller et al., 2006). Compared with culture, qPCR is more sensitive, specific and capable of accurate quantification for *M. catarrhalis* detection. The typing strategy based on mass spectrometric analysis of outer membrane proteins enables rapid and accurate differentiation of *M. catarrhalis* subpopulations and is well suited for large-scale sample testing. However, these two techniques are highly instrument-dependent, which limits their clinical application. Recently developed isothermal amplification coupled with lateral flow visual detection methods for *M*. *catarrhalis*, including multiple cross displacement amplification-fluorescent lateral-flow rapid test (MCDA-FRT, limit of detection: 35 fg per reaction, incubated at 65 °C for 40 min) and loop-mediated isothermal amplification-lateral flow biosensor (LAMP-LFB, limit of detection: 70 fg per reaction, incubated at 63 °C for 1 h), achieve rapid, simple and highly specific detection of target pathogen. Nevertheless, these two assays still possess obvious limitations: MCDA-FRT involves complex primer design and requires dedicated blue-light devices for fluorescence observation, whereas LAMP-LFB is restricted by multi-primer design complexity and relatively prolonged reaction time (Huang et al., 2024; Xiao et al., 2024). Currently, available detection methods for *M. catarrhalis* still cannot fully meet clinical needs for early and timely diagnosis. Therefore, there is an urgent need to establish a more accurate, rapid, and user-friendly auxiliary diagnostic method for *M. catarrhalis* infection. The development of such an assay is of great significance to facilitate on-site and point-of-care testing of this pathogen.

In 2006, Piepenburg et al. developed a novel isothermal amplification technique known as recombinase polymerase amplification (RPA) (Piepenburg et al., 2006). This method imposes significantly lower demands on instrumentation and enables much shorter reaction times. Owing to its simplicity, rapidity, efficiency, high sensitivity and specificity, RPA has been recognized as a promising nucleic acid detection alternative to conventional PCR (Li et al., 2020). Various detection strategies have been employed for RPA amplicons, including gel electrophoresis, real-time fluorescent quantification (exo-RPA), and lateral flow strip assay (RPA-LFS). The latter two approaches allow rapid, sensitive and specific detection, and thus represent the most widely used formats at present.

RPA-LFS combines isothermal RPA amplification and lateral-flow immunochromatography for visual detection. Target DNA is amplified at constant temperature, and labeled amplicons migrate on test strips to form visible bands via specific immuno-binding for result interpretation. The RPA-LFS assay has been extensively explored for the detection of bacterial pathogens due to its operational convenience, speed and high efficiency (Wang et al., 2022; Wang et al., 2025). For instance, Peng et al. established an RPA-LFS method for *Burkholderia pseudomallei* (*B. pseudomallei*) detection, which enabled visual readout within 30 min at a limit of detection as low as 25.6 copies (approximately equivalent to 512 CFU/mL) (Peng et al., 2019).Its sensitivity was comparable to that of qPCR, and no cross-amplification was observed with other species in the *B. pseudomallei* complex. RPA technology has also yielded promising applications in fungal detection. Wang et al. developed a duplex RPA-LFS system for *Candida glabrata* and *Candida krusei*, with detection limits of 10 copies/mL (approximately equivalent to 10 CFU/mL) and 100 copies/mL (approximately equivalent to 10 CFU/mL) in 30 min, respectively, exhibiting sensitivity comparable to qPCR (Wang et al., 2026).

The *copB* gene was widely used as a detection target for *M. catarrhalis* (Gadsby et al., 2015; Nawa et al., 2022), so we selected it as our target gene. In this study, an RPA-LFS assay targeting the *copB* gene of *M. catarrhalis* was established. As reported in the literature, bacterial culture combined with qPCR is a credible composite reference standard for performance assessment of assays targeting fastidious bacteria (Amaral et al., 2026). Considering that *M. catarrhalis* yields low positivity rates by culture, and that our qPCR assay has been thoroughly validated in prior publications (Greiner et al., 2003), we selected this combination for clinical performance evaluation. We systematically evaluated its sensitivity, specificity and clinical application value, with the ultimate aim of constructing a rapid, sensitive, specific and portable detection system for this pathogen.

## Materials and methods

### Strains and clinical samples

The strains used for the analytical sensitivity and specificity evaluation of RPA-LFS included *M. catarrhalis* ATCC 25238, 20 clinical strains of *M. catarrhalis*, and 22 other common pathogenic bacteria preserved in our laboratory (**Table 1**). ​A total of 300 specimens, including 255 sputum samples and 45 bronchoalveolar lavage fluid samples, were collected from hospitalized children suspected of *M. catarrhalis* pulmonary infection at Quanzhou Women’s and Children’s Hospital.​ DNA extraction was performed using the heat lysis method (Wang et al., 2022). For sputum specimens, 100 μL of sputum was mixed with 300 μL sterile ddH_2_O and vortexed vigorously to disperse mucus. For highly viscous sputum, an equal volume of 0.1 M DTT solution was added instead, followed by incubation at room temperature for 15 min for liquefaction. All treated sputum mixtures were centrifuged at 12 000 ×g for 3 min; the supernatant was discarded, and the pellet was resuspended in 100 μL sterile ddH_2_O. Pure bacterial suspension samples required no pre-treatment and proceeded directly to the subsequent lysis step. After thorough vortex mixing, the suspension was incubated at 100 °C for 10 min in a heat block to lyse bacterial cells and release genomic DNA. The lysate was cooled to room temperature and centrifuged at 12 000 ×g for 3 min. The supernatant containing crude genomic DNA was transferred into a new sterile tube and stored at −20 °C.

**Table 1:** Bacterial strains used in this study.

Species	Strain information	Quantity
*M. catarrhalis*	ATCC 25238	1
*M. catarrhalis*	Clinical isolates from deep sputum	20
*Escherichia coli*	Clinical isolate from blood	1
*Salmonella typhimurium*	Clinical isolate from stool	1
*Salmonella shigae*	Clinical isolate from stool	1
*Candida albicans*	Clinical isolate from secretion	1
*Streptococcus agalactiae*	Clinical isolate from blood	1
*Streptococcus pyogenes*	Clinical isolate from secretion	1
*Enterobacter cloacae*	Clinical isolate from blood	1
*Enterococcus faecalis*	Clinical isolate from urine	1
*Enterococcus faecium*	Clinical isolate from urine	1
*Pseudomonas aeruginosa*	Clinical isolate from sputum	1
*Staphylococcus aureus*	Clinical isolate from sputum	1
*Staphylococcus capitis*	Clinical isolate from blood	1
*Staphylococcus epidermidis*	Clinical isolate from blood	1
*Staphylococcus hominis*	Clinical isolate from blood	1
*Stenotrophomonas maltophilia*	Clinical isolate from sputum	1
*Streptococcus pneumoniae*	Clinical isolate from sputum	1
*Haemophilus influenzae*	Clinical isolate from sputum	1
*Klebsiella pneumoniae*	Clinical isolate from sputum	1
*Acinetobacter baumannii*	Clinical isolate from sputum	1
*Phytobacter diazotrophicus*	Clinical isolate from blood	1
*Klebsiella oxytoca*	Clinical isolate from sputum	1
*Proteus vulgaris*	Clinical isolate from urine	1

### Primers design and screening

Primers were designed based on the *copB* gene sequence from *M. catarrhalis* ATCC 25238 using Primer Premier 5.0 software. The minimum and maximum product sizes of the primers were set to 100 bp and 300 bp, respectively, while the minimum and maximum primer sizes were 30 bp and 35 bp, respectively. Primers with more than three consecutive base pairs in sequence pairing as well as more than one base at the 3’ end were discarded. The species specificity of the primer sequences was confirmed using Primer-Basic Local Alignment Search Tool (https://www.ncbi.nlm.nih.gov/tools/primer-blast/).

### RPA reaction

The RPA experiment was performed using the TwistAmp Liquid DNA Amplification Kit according to the manufacturer’s instructions. A 50 μL reaction mixture contained 25 μL of 2× reaction buffer, 5 μL of 10× mix, 2.5 μL of 20× core mix, 2.4 μL of 10 mM forward primer, 2.4 μL of 10 mM reverse primer, 9.2 μL of double-distilled water, 2.5 μL of 280 mM magnesium acetate, and 1 μL of template. The reaction tube was briefly centrifuged, and the reaction mixture was incubated at 37 °C for 30 minutes. Finally, the RPA amplification products were purified using the FastPure Gel DNA Extraction Mini Kit (Vazyme Biotech Co., Ltd., Nanjing, China). and separated by electrophoresis on a 1.5% agarose gel.

### Probe design and screening

Probes were designed using the online design tool (https://ezassay.com/primer?typeId=7&productId=82) with the following parameters: NFO probe type; probe length of 46–50 bp; GC content of 30%–70%; and a repeat nucleotide cut-off of 5 bp. All other parameters were set to their default values. To avoid false-positive results, mismatched bases were introduced into both the probe and reverse primer in this study to reduce primer-probe dimer formation. The principles for mismatch introduction were as follows: no more than 3 consecutive complementary bases between the probe and reverse primer; the complementary region between the probe and reverse primer did not cover the tetrahydrofuran (THF) site; no complementary pairing of more than 3 bases at the 3’ ends of the reverse primer and probe; and A-G and T-C mismatch combinations were prioritized. In addition, the 5’ end of the probe was labeled with FITC, the 3’ end was blocked with C3 spacer (SpC3), and a single base in the middle region of the probe was replaced with THF, following the rule that at least 30 bp of sequence was present before the THF site and 15 bp after the THF site. The 5’ end of the reverse primer was labeled with biotin. After the probe hybridizes to the target to form duplex DNA, the THF residue serves as the cleavage substrate of Nfo endonuclease (Endonuclease IV) supplied in the RPA-nfo kit. Cleavage at the THF site removes the 3’ terminal fragment blocked by SpC3 spacer, releasing a free 3’-OH. The deblocked probe is then converted into an effective primer for subsequent DNA polymerase extension.

### RPA-LFS assay

The RPA assay was performed using an RPA-nfo nucleic acid amplification reagent kit (Zhongce Biotechnology Co., Ltd, Hangzhou, China). Each 25 μL reaction system consisted of 12.5 μL of A buffer, 1.0 μL of forward primer (2 μM), 1.0 μL of reverse primer (2 μM), 0.3 μL of probe (2 μM), 6.95 μL of double-distilled water, 2 μL of DNA template, and 1.25 μL of B buffer.

First, according to the number of reactions, a mixture of A buffer, forward primer, reverse primer, probe, and double-distilled water was prepared in accordance with the reaction system, and the mixture was transferred into a detection unit tube containing lyophilized reaction powder. Subsequently, the DNA sample and B buffer were added sequentially. After thorough mixing, the reaction was incubated at 37 °C for 15 minutes. Then, 10 μL of the amplification product was mixed with 200 μL of sample buffer, and the LFS (Milenia Biotec GmbH, Gießen, Germany) was inserted into the mixture. The result was visually interpreted after standing for 3 minutes, completing the detection of *M. catarrhalis*.

### Analytical specificity and sensitivity of RPA-LFS

For analytical specificity testing, 20 clinical *M. catarrhalis* isolates and 22 other pathogenic microbial species were subjected to RPA-LFS amplification, and the results were visually observed and recorded. For analytical sensitivity assessment, 2 μL of DNA extracted from 10-fold serially diluted inactivated *M. catarrhalis* ATCC 25238 suspensions, covering a concentration range of 1.34×10^5^ to 1.34×10^0^ CFU/mL, was used as the template. To evaluate the potential interference of contaminating heterologous DNA, 1 μL of a heat-inactivated DNA mixture from 22 other pathogenic microbial species was added to the aforementioned RPA-LFS assays containing the serially diluted *M. catarrhalis* ATCC 25238 suspensions.

### Culture method

Three hundred clinical respiratory tract specimens were inoculated onto blood agar plates and chocolate agar plates, and incubated at 37 °C in a 5% CO_2_ incubator for 18–24 hours. Suspect colonies were picked, and *M. catarrhalis* was confirmed using VITEK MS (bioMérieux, France), a MALDI-TOF mass spectrometry platform that differentiates bacterial species by matching their characteristic ribosomal protein fingerprint profiles.

### PCR and qPCR method

PCR detection was used as the comparative reference method in this study. The PCR primers, PCR-copB-F and PCR-copB-R, for *M. catarrhalis* were synthesized according to sequences reported in the literature (Lim et al., 2021), as shown in **Table 2**. Conventional PCR was performed in accordance with the manufacturer’s instructions (Yeasen Biotechnology). The 50 μL reaction mixture contained 25 μL of 2× Hieff^®^ PCR Master Mix, 2 μL of forward primer (10 μM), 2 μL of reverse primer (10 μM), 1 μL of DNA template, and 20 μL of ddH_2_O. The PCR cycling conditions were as follows: initial denaturation at 95 °C for 3 min, followed by 40 cycles of denaturation at 95 °C for 30 s, annealing at 60 °C for 30 s, and extension at 72 °C for 30 s, with a final extension at 72 °C for 5 min. The amplification products were separated by electrophoresis on a 1.5% agarose gel.

**Table 2:** Primers and probes.

Primers/probe	Primer sequences (5’-3’)	Size (bp)
copB-F1	GGTGCAGGCTTATCAACCAACAAAGGTCATTC	32
copB-R1	TGTACCCTTTACCGCCTTTATAGTCGCTGTCA	32
copB-F2	TTTGGTAAAGCACAGACAGGATTTGGTCAGG	31
copB-R2	CACTTCTTGTAACCACATCATTGCCCAACAG	31
copB-P	FITC-TTTGACGAAGCTCAAGCAGGATTTGGTCAGGTA G[THF]TGCCCTTGTCTCTTA-C3 spacer	49
copB-R1B	Biotin-TGCACCCTCCACCGCCTTTATAGTCGCTGTCA	32
PCR-copB-F	ATTCGTGGCATGGGTCATAAT	21
PCR-copB-R	GTAACAATCGCACCRTTGGTT	20
q-PCR-copB-F	GTGAGTGCCGCTTTTACAACC	21
q-PCR-copB-R	TGTATCGCCTGCCAAGACAA	20
q-PCR-copB-P	HEX-TGCTTTTGCAGCTGTTAGCCAGCCTAA- BHQ1	27

F, forward primer; R, reverse primer; P, probe; red font indicates substituted bases.

qPCR in the present study was performed using the TaqMan probe method. The primer pair q-PCR-copB-F/q-PCR-copB-R and fluorogenic probe q-PCR-copB-P targeting the *copB* outer membrane protein gene of *M. catarrhalis* were designed as previously described in the literature (Greiner et al., 2003), as shown in **Table 2**. BeyoFast™ Probe qPCR Mix was purchased from Shanghai Beyotime Biotechnology Co., Ltd., Shanghai, China. The total qPCR reaction volume was 20 μL, consisting of 10 μL of 2× BeyoFast™ Probe qPCR Mix, 1 μL each of forward and reverse primers, 0.5 μL of probe, 2 μL of template DNA, and 5.5 μL of RNase-free water.The thermal cycling parameters were set as follows: initial denaturation at 95 °C for 2 min, followed by 40 cycles of denaturation at 95 °C for 15 s, annealing at 60 °C for 15 s, and final extension at 72 °C for 30 s. Fluorescence signals were collected at the annealing stage for Ct value analysis. Based on published criteria and our serial bacterial dilution verification, qPCR was defined positive at Ct ≤ 35 and negative at Ct > 35 (Almas et al., 2023). The limit of detection was determined to be 10^3^ CFU/mL, with a corresponding Ct value of 35.50 above the cutoff, (**Supplementary Figure 1**).

### Evaluation of clinical specimen detection based on RPA-LFS rapid detection technology

Four methods, namely RPA-LFS, qPCR, PCR and conventional culture, were used to detect 300 clinical specimens. Using the composite reference standard, the clinical sensitivity and specificity of the three molecular detection assays were calculated. Meanwhile, the positive detection rates among all four testing methods were compared statistically.

### Statistical analysis

Statistical analysis was performed using SPSS version 22.0 software. The chi-square test was used for enumeration data, and *P* < 0.05 was considered statistically significant.

## Results

### Screening of primers and probes

**Figure 1:**
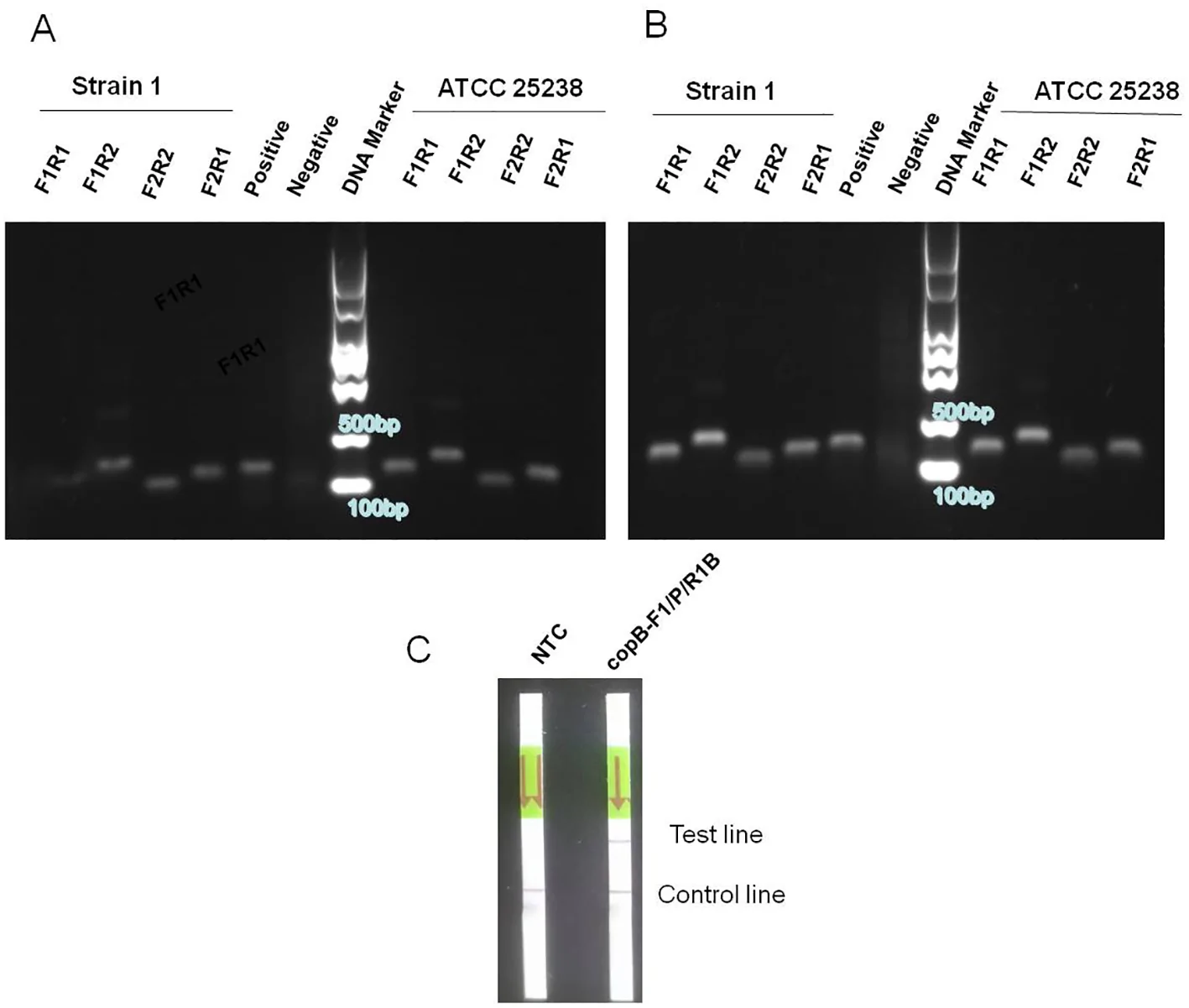
Results of primer pairs screening. **(A)** RPA amplification reaction performed at 39 °C for 30 min; **(B)** RPA amplification reaction performed at 37 °C for 30 min. The primer pair q-copB-F and q-copB-R was used for the positive control, while no template was added in the negative control. **(C)** RPA-LFS reaction using the primers combination of copB-F1,copB-P and copB-R1B performed at 37 °C for 15 min. NTC: No template control. 2 μL of *M. catarrhalis* ATCC 25238 DNA (10^4^ CFU/mL) was used as the template. The image represents the results of three independent experiments.

To screen the primers, primers copB-F1, copB-F2, copB-R1 and copB-R2 were first designed based on the conserved region of the *M. catarrhalis* target gene *copB* (**Table 2**). Genomic DNA from one clinical isolate strain and *M. catarrhalis* ATCC 25238 were then used as templates. Four primer pairs (F1R1, F1R2, F2R2 and F2R1) were used for RPA amplification at 39 °C and 37 °C for 30 minutes, respectively. Comparison between the gel electrophoresis images (**Figures 1A, B**) showed brighter bands in all reaction tubes in **Figure 1B**, indicating that the overall amplification efficiency was superior at 37 °C for 30 minutes. Further analysis of **Figure 1B** revealed non-specific bands in the F1R2 reaction tubes for both strains. Compared with the F2R2 and F2R1 reaction tubes, the target bands in the F1R1 reaction tubes were significantly brighter, demonstrating that the F1R1 primer pair achieved better amplification efficiency in both strains (**Figure 1**). Accordingly, the F1R1 primer pair was selected for subsequent experiments.

A suitable initial probe (TTTGGTAAAGCACAGACAGGATTTGGTCAGGTAGATGCCCTTGTCTCTTA) was selected to target the sequence between the forward F1 and reverse R1 primers. To avoid false-positive results, mismatched bases were introduced into the probe copB-P and reverse primer copB-R1B in this study to reduce the formation of dimers between the probe and the reverse primer. The substituted bases in the sequences of probe copB-P and reverse primer copB-R1B were indicated in bold and red, as shown in **Table 2**. The 5’ and 3’ ends of the probe copB-P were labeled with FITC and a blocking group SpC3, respectively, and the 35th base was replaced with THF. The 5’ end of primer copB-R1B was labeled with biotin. To verify the availability of the modified probe copB-P and reverse primer copB-R1B, an RPA-LFS assay was performed. As shown in the LFS results in **Figure 1C**, a distinct red test line was observed in the experimental group, whereas no test line appeared in the NTC, indicating the absence of false-positive signals.

### Evaluation of the analytical specificity of RPA-LFS

**Figure 2:**
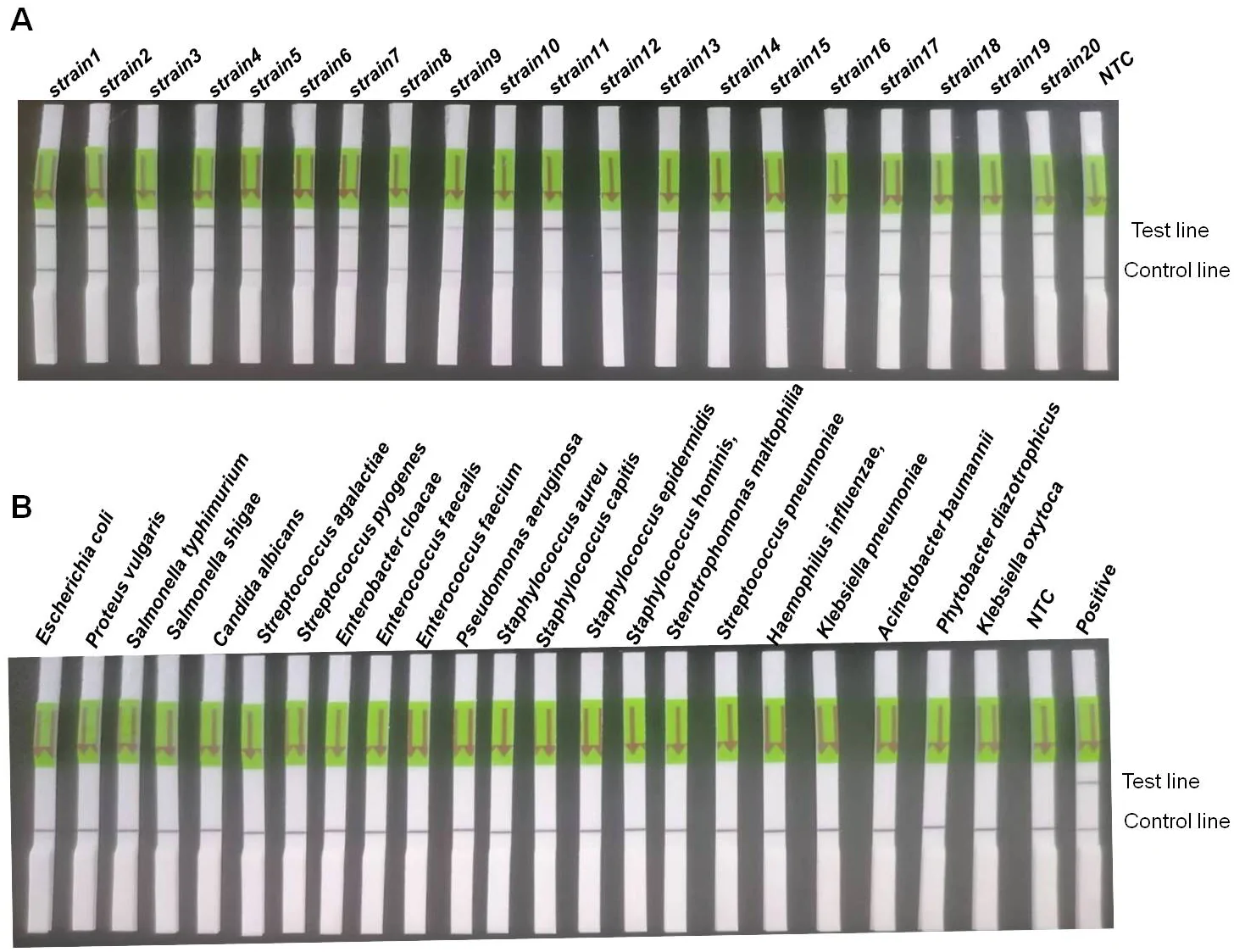
Specificity analysis of the RPA-LFS assay. **(A)** 2 μL heat-inactivated DNA from each of the 20 clinical *M. catarrhalis* isolates (1.34×10^5^ CFU/mL) was used as the template. NTC: No-template control. **(B)** 2 μL heat-inactivated DNA from each of the 22 other common pathogenic bacteria (1.34×10^5^ CFU/mL) was used as the template. NTC: No-template control.

To verify the analytical specificity of the established RPA-LFS assay, 20 clinically isolated *M. catarrhalis* strains were first tested using this system. LFS results demonstrated that all 20 strains yielded positive signals (**Figure 2A**). Furthermore, to confirm the assay specificity toward *M. catarrhalis*, 22 non-*M. catarrhalis* clinical isolates were examined, all of which tested negative in the LFS analysis (**Figure 2B**).

### Evaluation of the analytical sensitivity of RPA-LFS

**Figure 3:**
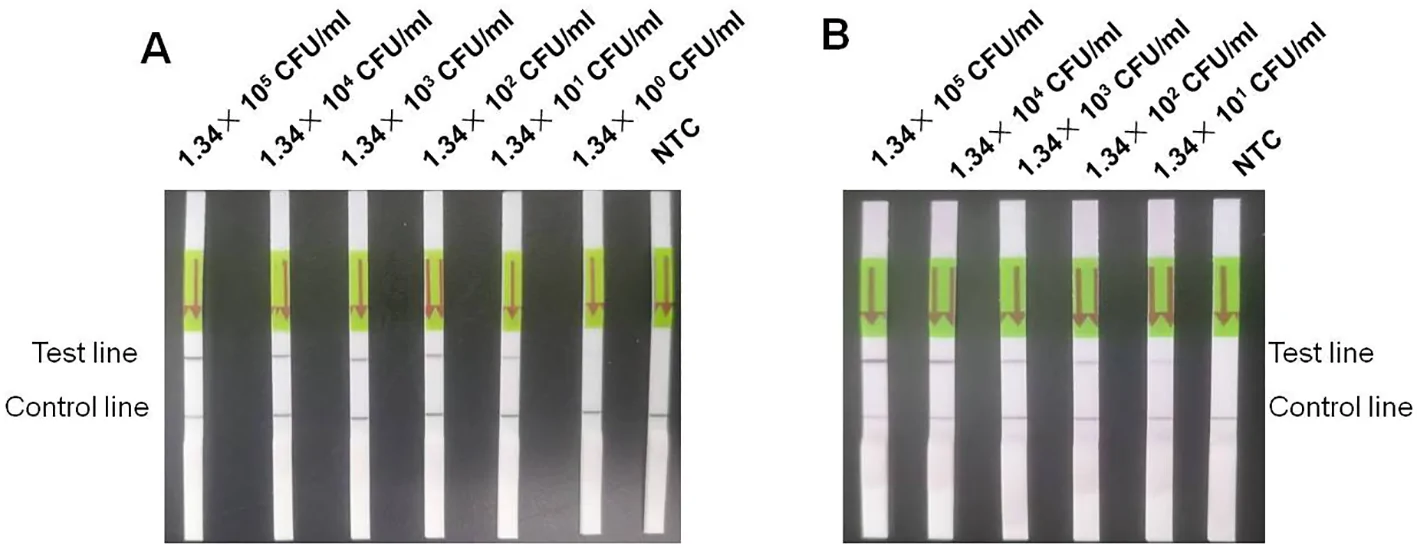
Validation of the limit of detection of RPA-LFS. **(A)** 2 μL DNA extracted from 10-fold serially diluted suspensions of inactivated *M. catarrhalis* ATCC 25238 was used as the template, with concentrations ranging from 1.34×10^5^ to 1.34×10^0^ CFU/mL. NTC: No-template control. **(B)** 1 μL DNA mixture extracted from heat-inactivated 22 non-*M. catarrhalis* strains was added to the reaction mixtures together with the serially diluted templates. NTC, No-template control.

To further analyze the detection sensitivity of the RPA-LFS system, *M. catarrhalis* at a concentration of 10^5^ CFU/mL was boiled and serially diluted, yielding a detection concentration range of 10^5^ to 10^0^ CFU/mL per reaction system. The LFS results in **Figure 3A** show that the limit of detection of RPA-LFS is 10^1^ CFU/mL. To assess the potential interference of heterologous DNA on assay sensitivity, a DNA mixture isolated from 22 non-*M. catarrhalis* strains was added to the reaction systems alongside the serially diluted templates. Notably, as shown in **Figure 3B**, the limit of detection remained unchanged in the presence of this non-specific DNA mixture, demonstrating that the RPA-LFS assay is robust against interference from other bacterial DNAs.

### Clinical performance evaluation of RPA-LFS

To verify the clinical application value of RPA-LFS, 300 clinical specimens were detected using RPA-LFS, qPCR, PCR, and conventional culture methods. The clinical sensitivity and specificity of RPA-LFS were 96.26% and 88.08%, respectively. Compared with qPCR, RPA-LFS exhibited comparable clinical sensitivity but significantly lower clinical specificity (**Table 3**). In comparison with conventional PCR, RPA-LFS achieved markedly improved clinical sensitivity accompanied by an obvious reduction in clinical specificity (**Table 3**). The differences in positive detection rates among the four methods were statistically significant (**Table 4**). Further pairwise comparison revealed that although the positive rate of RPA-LFS was numerically higher than that of qPCR, the difference was not statistically significant (*P*>0.05). Meanwhile, the positive detection rates of both RPA-LFS and qPCR were significantly higher than those of PCR and conventional culture, respectively (*P* < 0.05). Thus, the application of RPA-LFS for the detection of *M. catarrhalis* demonstrates high potential clinical value.

**Table 3:** Diagnostic performance of three molecular methods versus composite reference standard.

Methods		Culture+qPCR^a^		Sensitivity (%)	Specificity (%)
		Positive (n)	Negative (n)		
		107	193		
RPA-LFS	Positive (n)	103	23	96.26	88.08
	Negative (n)	4	170		
qPCR	Positive (n)	104	0	97.20	100
	Negative (n)	3	193		
PCR	Positive (n)	43	2	40.19	98.96
	Negative (n)	64	191		

^a^A composite reference standard consisting of culture and validated reference qPCR was used in the present study. Specimens were defined as true positive if culture was positive or qPCR was positive despite negative culture; samples negative in both culture and qPCR were considered true negative.

**Table 4:** Positive detection rates of four assays for clinical sputum specimens.

Methods	Positive number	Positive rate (%)	Chi-square test
RPA-LFS	126	42	Overall χ²= 117.2, *P* = 2.5212E-25
qPCR	104	34.66	
PCR	45	15	
Culture	27	9	

## Discussion

Based on the *copB*-targeted RPA-LFS system constructed in this study, we systematically evaluated its amplification performance, analytical sensitivity, analytical specificity and clinical diagnostic capacity. Herein, we elaborate on the rationality of this primer modification strategy, compare its detection performance with existing molecular diagnostic techniques, analyze the causes of inconsistent clinical testing results, and discuss the inherent limitations of this assay as well as directions for further optimization.

Dimers can be stably amplified, which may lead to false-positive signals. RPA can tolerate a certain degree of base mismatches without compromising amplification efficiency (Liu et al., 2019). Previous studies have also successfully established RPA-LFS assays by introducing mismatched bases (Yang et al., 2020; Wang et al., 2022). Similarly, in the present study, an RPA-LFS detection method was successfully developed by introducing mismatched bases into the probe and reverse primer. The RPA-LFS assay established in this study only requires 15 minutes of reaction time, which is shorter than the 20-minute reaction time reported in the literature (Wang et al., 2025; Chen et al., 2026).

With the high amplification efficiency of RPA and the user-friendly detection of LFS, the combined RPA-LFS assay has been successfully applied to point-of-care testing (POCT) for pathogens including SARS-CoV-2,*Staphylococcus haemolyticus* (Cao et al., 2022; Ji et al., 2023). The newly developed RPA-LFS assay for *M. catarrhalis* exhibits excellent detection analytical sensitivity and specificity. Wang et al. established an RPA-LFS assay for the detection of *Enterococcus faecium* and *Enterococcus faecalis*, with sensitivities of 10 CFU/mL and 10² CFU/mL, respectively, and a specificity of 100% (Wang et al., 2025). Similarly, the RPA-LFS assay established in this study for the detection of *M. catarrhalis* exhibits a limit of detection of approximately 10 CFU/mL, with an analytical specificity of 100%. The limit of detection of qPCR for *M. catarrhalis* was only 10^3^ CFU/mL (Greiner et al., 2003). This can explain why RPA-LFS yields a higher positive detection rate than qPCR in clinical specimens. Using culture combined with qPCR as the composite reference standard, the clinical specificity of RPA-LFS was only 88.08%. Samples with concentrations below 10^3^ CFU/mL frequently fail to generate measurable Ct values and are therefore classified as negative by qPCR, whereas RPA-LFS is able to identify these low-concentration specimens as positive. This discrepancy increases the apparent number of false positives and reduces the calculated clinical specificity of RPA-LFS. The newly developed RPA-LFS achieves clinical sensitivity equivalent to qPCR, constituting another prominent merit of this method. This assay delivers results within only 15 minutes without the use of large, sophisticated instruments, fully meeting the requirements for rapid clinical diagnosis. It exhibits excellent sensitivity and specificity when detecting clinical specimens. As a POCT approach, it is particularly suitable for primary medical institutions, remote areas and *M. catarrhalis*-endemic regions with limited laboratory facilities. Enabled by early and rapid diagnosis with this method, clinicians can formulate therapeutic regimens in a timely manner, thereby effectively reducing the disease burden caused by *M. catarrhalis* infections.

Furthermore, to avoid primer-base mismatching with target genes in clinical strains, we designed primers targeting the more conserved regions of the *copB* gene. The four false-negative cases of RPA-LFS may result from mutations located close to the 3’ terminus of the target binding site, preventing effective annealing between RPA primers and template DNA.

RPA-LFS is a lateral flow assay, and its results depend on naked-eye interpretation, which makes it susceptible to false-positive or false-negative results. In clinical strains, the newly developed RPA-LFS assay also demonstrates favorable detection rate and specificity. However, this method may exhibit cross-reactivity with other species within the genus *Moraxella* in clinical specimens, which warrants further in-depth investigation.

## Conclusion

The novel RPA-LFS assay established in this study for *M. catarrhalis* is time-saving, instrument-low-dependent, and exhibits high clinical sensitivity and specificity, demonstrating promising potential for clinical application. In conclusion, the newly developed RPA-LFS method can serve as another important tool for the clinical detection of *M. catarrhalis*.

## Data Availability

The original contributions presented in the study are included in the article/**Supplementary Material**. Further inquiries can be directed to the corresponding author.

## Supplementary material

Supplementary Figure 1 Verification of the limit of detection of the qPCR assay.
